# Differentiation of small (≤ 3 cm) hepatocellular carcinomas from benign nodules in cirrhotic liver: the added additive value of MRI-based radiomics analysis to LI-RADS version 2018 algorithm

**DOI:** 10.1186/s12876-021-01710-y

**Published:** 2021-04-07

**Authors:** Xi Zhong, Tianpei Guan, Danrui Tang, Jiansheng Li, Bingui Lu, Shuzhong Cui, Hongsheng Tang

**Affiliations:** 1 Department of Medical Imaging, Affiliated Cancer Hospital and Institute of Guangzhou Medical University, Guangzhou, 510095 China; 2 Department of Abdominal Surgery, Affiliated Cancer Hospital and Institute of Guangzhou Medical University, No.78, Hengzhigang Rd, Guangzhou, 510095 China

**Keywords:** Hepatocellular carcinoma, Liver cirrhosis, Magnetic resonance imaging, Diagnosis

## Abstract

**Background:**

Accurate characterization of small nodules in a cirrhotic liver is challenging. We aimed to determine the additive value of MRI-based radiomics analysis to Liver Imaging Reporting and Data System version 2018 (LI-RADS v 2018) algorithm in differentiating small (≤ 3 cm) hepatocellular carcinomas (HCCs) from benign nodules in cirrhotic liver.

**Methods:**

In this retrospective study, 150 cirrhosis patients with histopathologically confirmed small liver nodules (HCC, 112; benign nodules, 44) were evaluated from January 2013 to October 2018. Based on the LI-RADS algorithm, a LI-RADS category was assigned for each lesion. A radiomics signature was generated based on texture features extracted from T1-weighted, T2W, and apparent diffusion coefficient (ADC) images by using the least absolute shrinkage and selection operator regression model. A nomogram model was developed for the combined diagnosis. Diagnostic performance was assessed using receiver operating characteristic curve (ROC) analysis.

**Results:**

A radiomics signature consisting of eight features was significantly associated with the differentiation of HCCs from benign nodules. Both LI-RADS algorithm (area under ROC [A_z_] = 0.898) and the MRI-Based radiomics signature (A_z_ = 0.917) demonstrated good discrimination, and the nomogram model showed a superior classification performance (A_z_ = 0.975). Compared with LI-RADS alone, the combined approach significantly improved the specificity (97.7% vs 81.8%, *p* = 0.030) and positive predictive value (99.1% vs 92.9%, *p* = 0.031) and afforded comparable sensitivity (97.3% vs 93.8%, *p* = 0.215) and negative predictive value (93.5% vs 83.7%, *p* = 0.188).

**Conclusions:**

MRI-based radiomics analysis showed additive value to the LI-RADS v 2018 algorithm for differentiating small HCCs from benign nodules in the cirrhotic liver.

**Supplementary Information:**

The online version contains supplementary material available at 10.1186/s12876-021-01710-y.

## Background

Early detection of hepatocellular carcinoma (HCC) is the only chance for effective treatment and long-term survival in high-risk patients. However, hepatocarcinogenesis in cirrhosis usually shows a multistep progression from benign nodules to small HCCs (≤ 3 cm), and finally, overt progressive HCC. Accurate characterization of small HCCs and benign nodules is challenging due to the overlap of imaging features during the hepatocarcinogenesis process [1, 2]. To standardize terminology and criteria for interpreting and reporting the imaging results of the liver, Liver Imaging Reporting and Data System (LI-RADS) was established by the American College of Radiology. The initial version of LI-RADS was published in 2011, with major updates released in 2014, 2017, and 2018 [3–7]. LI-RADS reflects the relative probability of HCC development by assigning categories ranging from LR-1 to LR-5 (definitely HCC) or LR-TIV (definite tumor in vein) based on the presence of specific imaging features [5, 6].

Recently, the LI-RADS algorithm has been widely used to characterize liver nodules in patients with a high risk of HCC. The LI-RADS algorithm comprises categories based on major features, and ancillary features are used to improve characterization and detection, promote confidence, or modify the LI-RADS category after the involvement of ancillary features [5]. Regarding the performance of LI-RADS for diagnosing small HCCs, LR-5/LR-TIV categories showed fairly high specificity but limited sensitivity; on the contrary, combining the LR-4 and LR-5/LR-TIV categories for diagnosing HCC markedly improved sensitivity but led to a reduction in specificity [8–11]. Particularly, LI-RADS, which is based on the identification of some categories of liver lesions by means of a conceptual and non-quantitative probability approach, has many limitations [8]. Thus, it is necessary to seek a noninvasive and quantitative method for identifying these small cirrhotic nodules.

Radiomics is a promising tool that allows for extracting numerous quantitative parameters by converting imaging data into a high-dimensional mineable feature set with a series of data-characterization algorithms. Regarding differential diagnosis in oncology, MRI-based radiomics has afforded encouraging results in the classification of primary breast tumor [12, 13], differentiation of the primary site of origin of brain metastases [14], identification of adrenal metastases from adrenal adenomas [15], and differentiation of benign and malignant prostate nodules [16, 17]. For liver assessments, MRI-based radiomics can be applied to differentiate hemangiomas, metastases, and HCCs [18], or differentiate between cysts and hemangiomas [19].

To our knowledge, the added value of MRI-based radiomics to the LI-RADS algorithm in the characterization of cirrhotic nodules is still undefined. We speculated that MRI-based radiomics combined with LI-RADS may overcome some of the limitations of LI-RADS and improve the diagnostic efficacy. Thus, the purpose of this study was to explore the additive value of MRI-based radiomics to the LI-RADS v 2018 algorithm for the differentiation of small HCCs from benign nodules.

## Materials and methods

### Patients

**Fig. 1:**
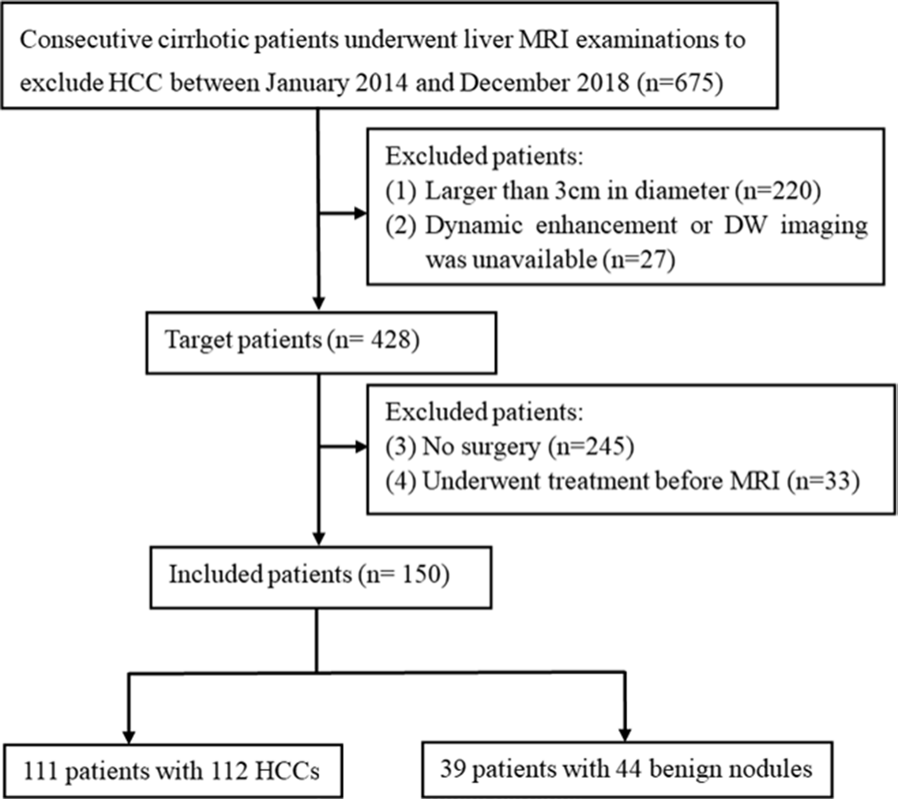
Flowchart of the study population

This retrospective study was approved by the institutional review board of Affiliated Cancer Hospital & Institute of Guangzhou Medical University. From January 2013 to October 2018, we reviewed liver MRI, clinical, and pathology data of 675 consecutive cirrhosis patients. The following patients were included: (1) patients with at least one nodule having a diameter smaller than or equal to 3 cm; (2) patients who had undergone dynamic enhancement and diffusion-weighted (DW) imaging; (3) patients in whom pathological confirmation by surgical resection had been performed; and (4) patients who did not undergo any treatment before MRI. Subsequently, 525 patients were excluded due to the following reasons: (1) presence of a nodule with a diameter larger than 3 cm (n = 220); (2) unavailability of dynamic enhancement or DW imaging data (n = 27); (3) lack of pathological data (n = 245); and (4) receipt of treatment prior to MRI (n = 33). Finally, 111 patients with 112 HCCs and 39 patients with 44 benign nodules were included. The patient inclusion flowchart is shown in Fig. 1.

### Image acquisition

Sixty-eight patients underwent gadoxetic acid-enhanced MRI (Gd-EOB-MRI) and 82 patients underwent gadopentetate dimeglumine-enhanced (Gd-DTPA) MRI. MR images were obtained using a 3.0-T whole-body MR system (Achieva; Philips Healthcare) with a 16-channel phased-array coil. Scanning sequences included a dual gradient-recalled echo T1-weighted sequence, an axial T2-weighted fat-suppression (FS) turbo spin-echo (TSE) sequence, dynamic contrast-enhanced MRI-Gd-EOB-MRI (unenhanced, arterial [20–35 s], portal [60 s], transitional phase [3 min], and hepatobiliary phase [20 min]) or Gd-DTPA-MRI (unenhanced, arterial [20–35 s], portal [60 s], and equilibrium [3 min]), and DW imaging with b-values of 0 and 800 s/mm^2^. Apparent diffusion coefficient (ADC) maps were created automatically on a voxel-by-voxel basis from the two b-values. The detailed MRI parameters are summarized in Table 1.

**Table 1:** MRI sequences and parameters

Sequence	FS	TR/TE (ms)	FA	ST (mm)	FOV (cm)	Matrix
*T1-w dual gradient recalled echo*						
In-phase	No	10/2.5	10°	5	30–38	256 × 224
Opposed-phase	No	10/3.55	10°	5	30–38	256 × 224
Breath-hold FS T2-w	Yes	2096/72	90°	5	30–38	324 × 256
DWI	Yes	1600/70	90°	5	30–35	100 × 100
T1-w dynamic enhanced	Yes	3.1/1.5	10°	2	32–38	228 × 211

*FS* fat suppression, *TR* repetition time, *TE* echo time, *FA* flip angle, *ST* slice thickness, *FOV* field of view, *T1-w* T1 weighted, *T2-w* T2 weighted

### Qualitative image analysis

The radiologists were informed that this study attempted to evaluate the contribution of LI-RADS v 2018 in HCC detection but they were blinded to the patients’ clinical data and pathologic diagnosis. Two radiologists (observer 1, JSL, with 15 years of experience; and observer 2, BGL, with 10 years of experience) independently analyzed all MR images for assessing major and ancillary features, and assigned a LI-RADS category for each lesion. All disagreements on LI-RADS categories were solved by consensus 1 month after the individual interpretations.

First, LI-RADS categories were assigned based on major features (Supplementary Table 1) and the observations were categorized as LR-3, LR-4, and LR-5 [5, 6]. The growth threshold was eliminated from the assessment, because follow-up assessments for more than 6 months were performed in only 10 patients. Second, the radiologists were requested to upgrade or downgrade the final LI-RADS categories based on the presence of ancillary features (Supplementary Table 2). The rules for application of ancillary features to adjust LI-RADS categories assigned by major features were based on the criteria in LI-RADS v 2018 [5]. Finally, LI-RADS categories based on the combination of major and ancillary features were documented for each lesion assessed.

**Table 2:** Characteristics of patients and lesions

Parameters	HCCs	Benign nodules	*P* value
*Patient-wise analysis*			
Number	111	39	
Age, median [range] (years)	55 [35–81]	59 [41–82]	0.198
Male/female	97/14	34/5	0.706
Child–Pugh			NA
A	56	19	
B	45	14	
C	10	6	
AFP^a^			
Patients with high AFP serum^b^	45	11	NA
Patients with AFP serum > 200 ng/ml	24	2	NA
Etiology of liver cirrhosis^c^			NA
HBV	86	34	
HCV	18	3	
Ethanol	20	6	
Number of nodules/patient			NA
One nodule	110	34	
Two nodules	1	5	
*Lesion-wise analysis*			
Number	112	44	NA
Histopathologic feature of lesions			
Well-differentiated HCC	47	0	
Moderately/Poorly differentiated HCC	75	0	NA
Dysplastic nodule	0	39	
Regenerative nodule	0	5	
Nodule size, median [range] (cm)	2.1 [0.9–3.0]	1.7 [0.6–2.9]	0.027

Continuous variables are expressed as a median/range and qualitative variables as the total count

*NA* not assessment*, AFP* alpha-fetoprotein

^a^43 missing data

^b^High AFP serum means above the upper normal limit

^c^A patient could have multiple etiologies

## Radiomics analysis

### Feature extraction

**Fig. 2:**
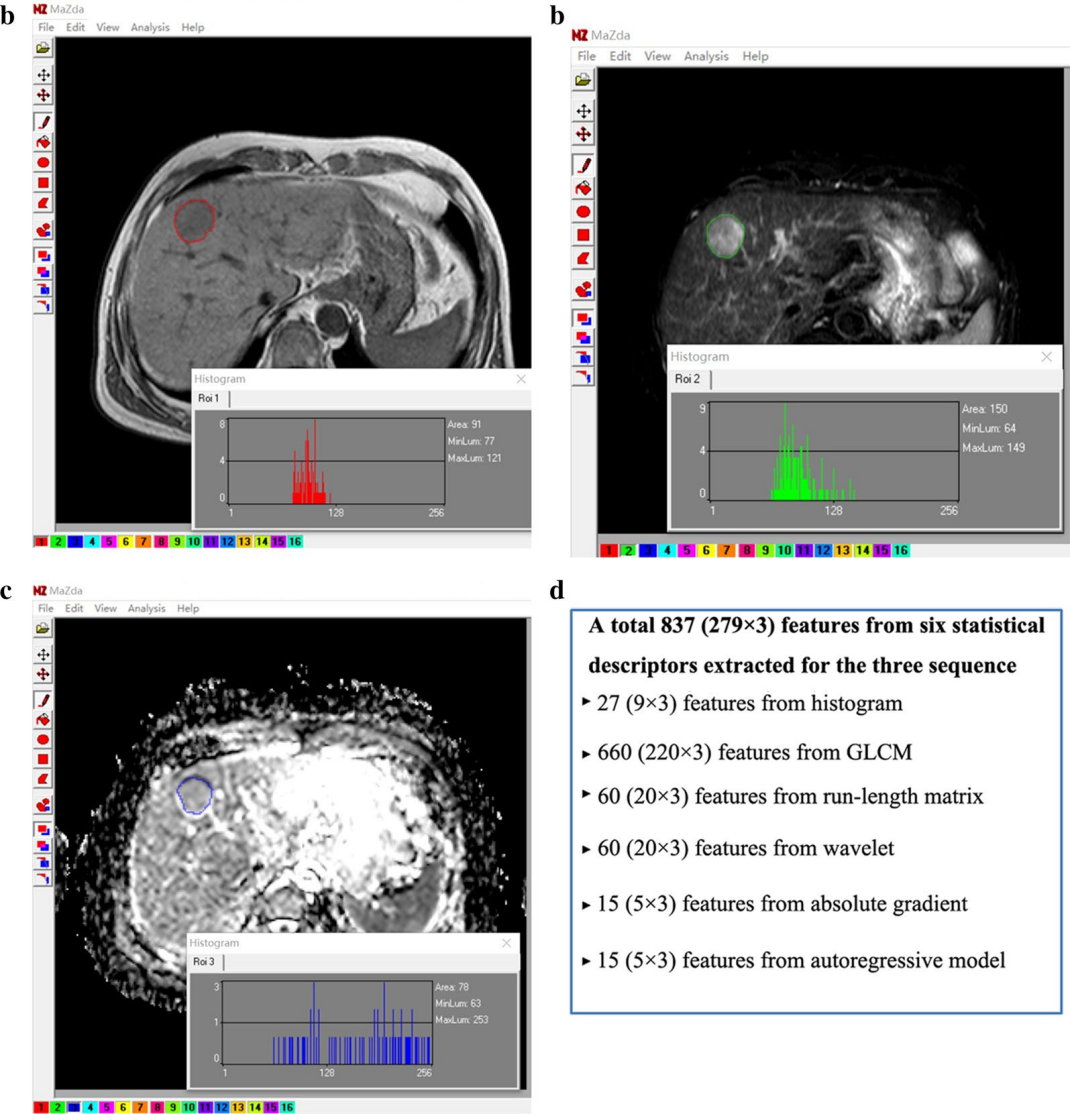
Extraction of image segmentation and texture features. Using the software package MaZda 4.6 for texture calculation for a 78-year-old man with a pathologically proven HCC, regions of interest (ROIs) were manually delineated in the largest cross-sectional area of the lesion in the in phase T1W image (**a**), FS-T2W image (**b**), and ADC maps (**c**). A total of 279 quantitative texture features from six statistical image descriptors were extracted for each ROI, thus a total of 837 features based on the three sequences were determined for each lesion (**d**)

Axial in-phase T1-WI, fat-suppression (FS) T2-WI, and ADC maps in the “.dicom” format were imported to MaZda 4.6 (http://www.eletel.p.lodz.pl/programy/mazda/) for texture feature extraction. Two radiologists (XZ and BGL, with 5 and 10 years of experience in medical image segmentation) manually drew a region of interest (ROI) for each nodule on the image section that depicted the maximum area (Fig. 2a–c). To minimize the influence of contrast and brightness variation, the ROI gray level was normalized [19–21], after which 279 texture features resulting from six statistical image descriptors were extracted for each ROI, thus a total of 837 features based on the three sequences were determined for each lesion (Fig. 2d). The detailed feature names and numbers are summarized in Supplementary Table 3.

**Table 3:** Frequencies of LI-RADS categories based on major and ancillary features stratified by observers

LI-RADS categories	Observer 1		Observer 2		Consensus	
	HCCs	Benign nodules	HCCs	Benign nodules	HCCs	Benign nodules
LR-2	0 (0%)	5 (11.4%)	0 (0%)	6 (13.6%)	0 (0%)	5 (11.4%)
LR-3	7 (6.3%)	31 (70.5%)	9 (8.0%)	29 (65.9%)	7 (6.3%)	31 (70.5%)
LR-4	64 (57.1%)	6 (13.6%)	60 (53.6%)	7 (15.9%)	63 (56.3%)	6 (13.6%)
LR-5	40 (35.7%)	2 (4.5%)	43 (38.4%)	2 (4.6%)	42 (37.5%)	2 (4.5%)
LR-TIV	1 (0.9%)	0 (0%)	0 (0%)	00 (0%)	0 (0%)	0 (0%)
Total	112	44	112	44	112	44

Data are expressed as numbers of lesions. *LI-RADS* liver imaging reporting and data system, *TIV* definite tumor in vein

### Radiomics signature construction

To determine the discriminative texture features for differentiating HCCs from benign nodules, first, feature selection was performed based on reproducibility and redundancy with reference to previous studies [22–24]. Texture features with interclass correlation coefficients (ICC) values ≥ 0.80 were identified as highly reproducible features and remained for further selection. Second, we performed feature selection from the remaining dataset by using the Mann–Whitney U test, and features with a P-value less than 0.05 were maintained. Finally, a radiomics signature was constructed by using the least absolute shrinkage and selection operator (LASSO) logistic regression analysis with tenfold cross-validation based on minimum criteria [25, 26].

With the combination of LI-RADS and radiomics signature, a radiomics nomogram model was constructed. A calibration curve was drawn to appraise the calibration of the radiomics nomogram, accompanied by the Hosmer–Lemeshow test to assess the goodness-of-fit of the nomogram.

### Statistical analysis

All statistical analyses were performed using R software (version 3.5.3, http://www.rproject.org/) and SPSS 16.0 (SPSS Inc., Chicago, IL, USA) software package, and statistical significance was set at *P* < 0.05. LASSO logistic regression was performed using R statistical software with the "glmnet" package. The nomogram and calibration plots were created using the "rms" package, and the Hosmer–Lemeshow test was conducted using the "generalhoslem" package. Other statistical analyses were performed using SPSS 16.0; inter-reader variability between the two observers for LI-RADS categories was appraised using kappa statistics. The diagnostic performance for each diagnosis model was assessed using by receiver-operator characteristic curve (ROC) analysis. The Mann–Whitney U test and Pearson chi-square test (or Fisher test) were used for continuous and categorical variables, respectively.

## Results

### Patient characteristics

Of the 150 patients, 111 patients (74%) with 112 nodules were diagnosed as having HCC (diameter range 0.9–3.0 cm; median, 2.1 cm), and 105 nodules were confirmed by resection, while 7 nodules were confirmed by aspiration biopsy. Thirty-nine patients with 44 nodules were diagnosed as showing benign nodules (diameter range 0.6–2.9 cm; mean, 1.7 cm), of which 32 nodules were confirmed by resection and 12 nodules were confirmed by aspiration biopsy. There was a significant difference in nodule diameter between HCCs and benign nodules. Of the 21 patients with HCV infection (18 HCCs, 3 benign nodules), 17 patients (14 HCCs, 3 benign nodules) received antiviral therapies; of the 110 patients with HBV infection (86 HCCs, 34 benign nodules), 62 (35 HCCs, 27 benign nodules) received antiviral therapies. The proportion of patients who received antiviral therapies in the HCC group was lower than that of benign nodule group: 77.8% (14/18) vs. 100% (3/3) for HCV patients, and 40.7% (35/86) vs 79.4% (27/34) for HBV patients. The detailed patient and lesion characteristics are summarized in Table 2.

### Performance of the LI-RADS v 2018 algorithm

The frequencies of LI-RADS categories based on the combination of major and ancillary features in assessments by the two observers and the consensus reports are shown in Table 3. Inter-observer agreement in the assessment of LI-RADS categories was very good (k = 0.910). When LI-RADS categories were used in consensus for differentiation of sHCC from benign nodules, in the ROC analysis, with a cut off value ≥ LR-4, the LI-RADS v 2018 algorithm demonstrated an Az of 0.898 (95% CI: 0.834, 0.961), sensitivity of 93.8% (105/112), specificity of 81.8% (36/44), positive predictive value (PPV) of 92.9% (105/113), negative predictive value (NPV) of 83.7% (36/43), and accuracy of 90.4% (141/156).

### Performance of MRI-based radiomics analysis

**Fig. 3:**
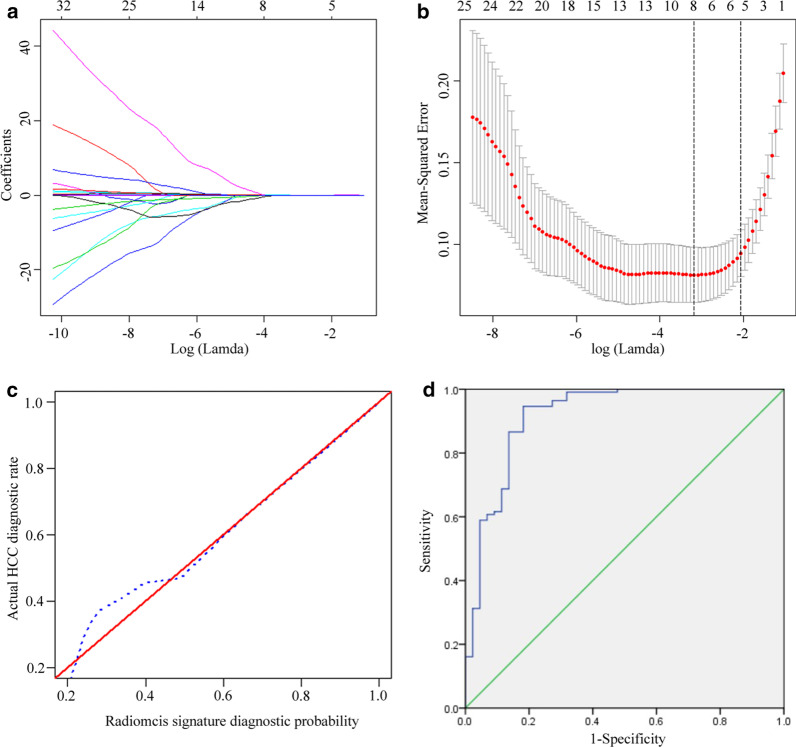
Radiomics signature development and diagnostic efficiency assessment. A radiomics signature was obtained using the LASSO algorithm, and the optimal tuning parameter (Lambda) in the LASSO model was selected using tenfold cross-validation based on minimum criteria. **a** LASSO coefficient profiles of the texture features. **b** The optimal values of log (Lambda) =  − 3.126 and eight non-zero coefficients were chosen (vertical line). **c** Calibration curves of the radiomics signature, the 45° red lines represent a perfect prediction, and the dotted blue lines represent the predictive performance of the radiomics signature; the closer the dotted blue line fit is to the red line, the better the predictive accuracy of the radiomics signature is. **d** Diagnostic efficiency of radiomics signature using ROC analysis

Of these 837 features, 301 features with ICC values ≥ 0.80 were selected for further reduction, of which 57 texture parameters with *p *values less than 0.05 by using Mann–Whitney U test remained for subsequent LASSO analysis, and these features measured by the two radiologists were averaged. A radiomics signature consisting of eight features with non-zero coefficients that were significantly associated with the differentiation of HCCs from benign nodules was generated by using the LASSO logistic regression model (Fig. 3a, b), and showed good calibration (Fig. 3c). Details regarding the features and their coefficients are shown in Table 4. The radiomics score for each lesion was calculated by using a formula resulting from the eight features weighted by their coefficients. Based on the radiomics scores, ROC analysis (Fig. 3d) showed that the radiomics signature yielded an Az of 0.917 (95% CI: 0.860, 0.974), sensitivity of 93.8% (105/112), specificity of 86.4% (38/44), PPV of 94.6% (105/111), NPV of 84.4% (38/45), and accuracy of 91.7% (143/156).

**Table 4:** Calculation formula for radiomics signature

Parameters	Textural groups	Coefficients
Intercept		− 1.01
T1W-Vertl_RLNonUni	Run-length matrix	− 0.0010
T1W-S(5,-5)DifVarnc	GLCM	0.0019
T2W-S(5,5)SumOfSqs	GLCM	0.0024
T2W-WavEnLL_s-1	Wavelet	0.0044
T2W-S(0,1)SumEntrp	GLCM	0.0751
T2W-S(3,-3)SumOfSqs	GLCM	0.2208
T2W-Sigma	Autoregressive model	0.0109
ADC-S(1,0)SumVarnc	GLCM	− 0.0295

*Vertl_RLNonUni* vertical run-length nonuniformity, *DifVarnc* difference variance, *SumOfSqs* sum of squares, *WavEnLL_s-1* wavelet energy LL scale1, *SumEntrp* sum entropy, *SumVarnc* sum variance

### Added value of radiomics analysis to LI-RADS v 2018 algorithm

**Fig. 4:**
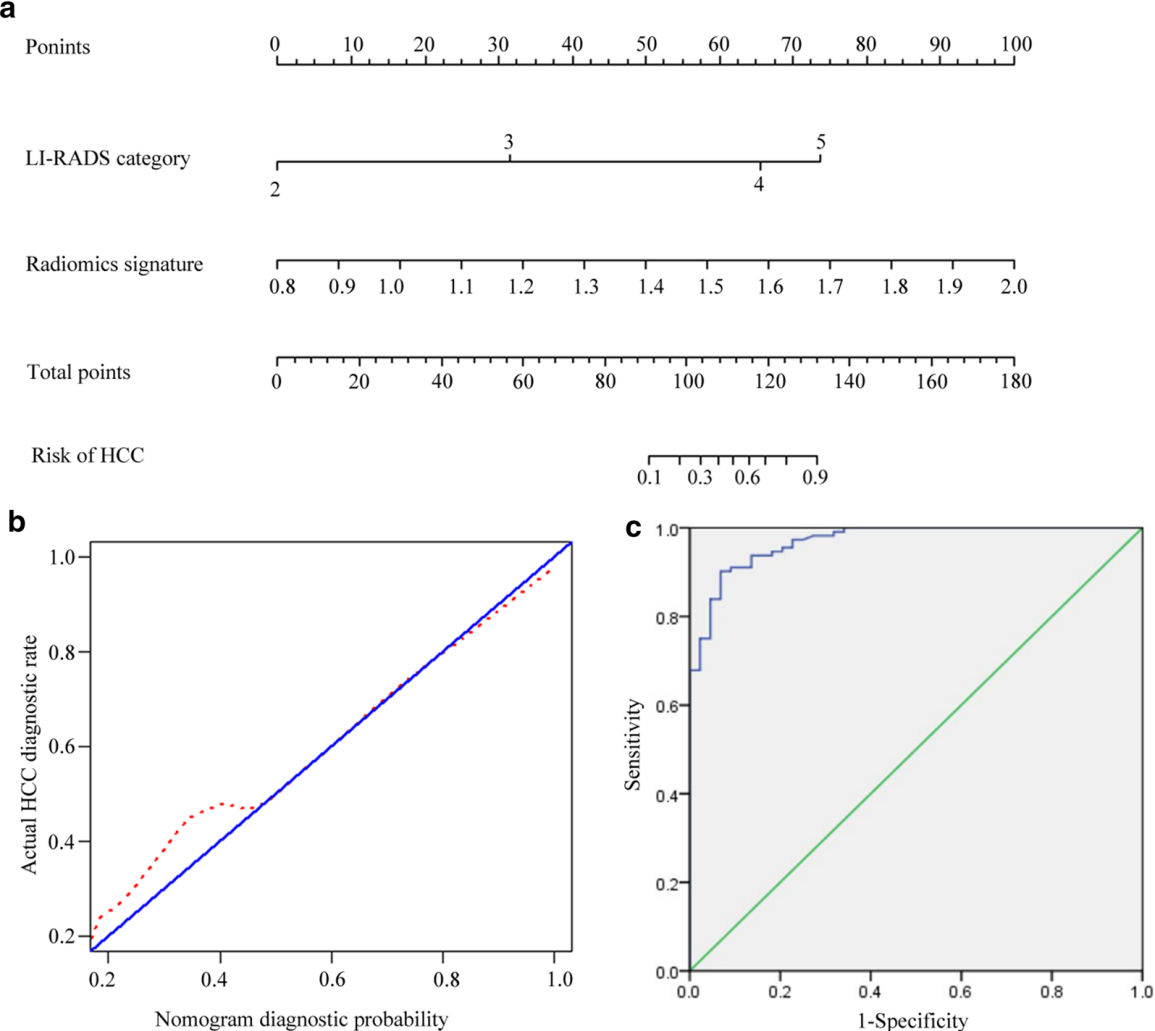
Radiomics nomogram construction, calibration, and performance assessment. **a** The LI-RADS categories and radiomics signature were combined, and a radiomics nomogram was plotted. **b** Calibration curves of the radiomics nomogram, the 45° blue lines represent a perfect prediction, and the dotted red lines represent the predictive performance of the radiomics signature. **c** ROC analysis showed that the **c**ombined radiomics nomogram demonstrated superior classification performance

The detailed performance parameters for each diagnostic pattern are summarized in Table 5. For combined diagnosis, a radiomics nomogram model that included the radiomics signature and LI-RADS categories was established (Fig. 4a) and showed good calibration (Fig. 4b). Using ROC analysis (Fig. 4c), the radiomics nomogram demonstrated a superior Az value of 0.975 (0.954–0.996) than that of LI-RADS. In comparison with LI-RADS alone, the radiomics nomogram model showed a significant improvement in specificity (97.7% vs 81.8%, *p* = 0.030), PPV (99.1% vs 92.9%, *p* = 0.031), and accuracy (97.4% vs 93.8%, *p* = 0.016), and a comparable sensitivity (97.3% vs 93.8%, *p* = 0.215) and NPV (93.5% vs 83.7%, *p* = 0.188).

**Table 5:** Diagnostic performance of LI-RADS categories in the discrimination of HCCs from benign nodules

Diagnostic pattern	A_z_ (95%CI)	Sensitivity	Specificity	PPV	NPV	Accuracy
LI-RADS	0.898 (0.834–0.961)	93.8% (105/112)	81.8% (36/44)	92.9% (105/113)	83.7% (36/43)	90.4% (141/156)
Radiomics signature	0.917 (0.860–0.974)	93.8% (105/112)	86.4% (38/44)	94.6% (105/111)	84.4% (38/45)	91.7% (143/156)
Combined nomogram	0.975 (0.954–0.996)	97.3% (109/112)	97.7% (43/44)	99.1% (109/110)	93.5% (43/46)	97.4% (152/156)

*LI-RADS* liver imaging reporting and data system, *A*_*z*_ area under the receiver operating characteristic curve, *PPV* positive predictive value, *NPV* negative predictive value

## Discussion

In the present study, we compared cirrhotic nodule classification using LI-RADS v 2018 alone and a combination of LI-RADS v 2018 and MRI-based radiomics analysis. We evaluated the added value of the MRI-based radiomics analysis in sHCC diagnosis. By combining LI-RADS and radiomics analysis, we constructed a radiomics nomogram model, and we observed improved lesion classification performance (Az: 0.975) than that achieved with the LI-RADS algorithm alone (Az, 0.898). Particularly, in comparison with the LI-RADS algorithm alone, the nomogram demonstrated a significant improvement in specificity and PPV, with comparable sensitivity and NPV.

LI-RADS is widely applied in the characterization of cirrhotic nodules, and the LI-RADS algorithm based on a combination of major features and ancillary features has shown superior classification performance over approaches using major features alone, with previous studies indicating that the addition of ancillary features increased sensitivity while preserved the specificity for HCC [8–11]. In this study, we assessed the performance of the LI-RADS v 2018 algorithm based on the combination of major features and ancillary features in the differentiation of sHCC from benign nodules. ROC analysis showed that the cut-off value for LI-RADS category was ≥ LR-4, and this result was associated with the finding that approximately half of the HCCs were categorized as LR-4 category. Using LR-4 and LR-5/LR-TIV categories as the criteria for diagnosing HCC, we found that the LI-RADS v 2018 algorithm yielded an overall accuracy of 90.4%. Our results are consistent with those of several previous studies, which indicated that the combination of LR-4 and LR-5/LR-TIV categories demonstrated better diagnostic performance than that of LR-5/LR-TIV categories [5, 6, 8, 9].

Radiomics analysis provides quantitative texture features that may be associated with the histopathological characteristics of lesions, and radiomics thus shows promising resolving power in differentiation of liver benign and malignant diseases [18], classification of hepatic fibrosis and cirrhosis of various grades [27], or prediction of the histological grading of HCC [28]. In this study, we assessed the potential value of multi-parametric MRI-based radiomics analysis for distinguishing small HCCs from benign nodules in cirrhosis. We found that the radiomics signature based on MRI texture features demonstrated a slightly better classification performance than that of the LI-RADS v 2018 algorithm. Actually, in comparison with the LI-RADS v 2018 algorithm, MRI-based radiomics analysis showed equal sensitivity of 93.8%, and a higher specificity of 86.4%. The results supported our previous findings in which MRI-based texture analysis produced a greater performance than qualitative diagnosis with Gd-EOB dynamic MRI or DWI [29]. In addition, the classification accuracy of 91.7% with MRI-based radiomics analysis in this study was also similar with the previously reported values of 84.5–92% in studies where MRI-based texture analysis was used to distinguish metastases and HCCs [18], HCCs and benign hepatocellular tumors [30], or cysts and hemangiomas [19]. As mentioned in previous studies [31, 32], accurate identification and classification of HCC substages demonstrated great clinical value in the assessment of untreated HCC patients. The potential application value of MRI-based radiomics in classifying HCC substages needs to be studied further.

In this study, most of the differentiation-related features selected were derived from T2WI, supporting a previous study where T2WI-based texture analysis produced better overall accuracy than T1-weighted images in discrimination of liver cysts and hemangiomas [19]. However, unlike previous studies in which texture features based on ADC maps showed more discriminative power than T2WI features in classification of benign and malignant prostatic nodules [33], we found that only one feature based on ADC maps was selected to the radiomics signature. This difference might be partially explained by the fact that the cirrhotic parenchyma shows lower ADC values compared to normal hepatic parenchyma due to the abundance of proton-poor fibrotic tissue and the decreased blood flow, resulting in a restricted value of ADC maps for distinguishing nodules in cirrhotic liver [34, 35].

The most important innovation of this study was that we assessed the additive value of MRI-based radiomics analysis to LI-RADS v 2018 algorithm in differentiation of sHCC from benign nodules. Compared with LI-RADS alone, with the addition of radiomics analysis, the radiomics nomogram model showed a significant improvement in overall accuracy (97.4% vs 90.4%), specificity (97.7% vs 81.8%) and PPV (99.1% vs 92.9%). Furthermore, the sensitivity increased from 93.8% to 97.3%, and NPV increased from 83.7% to 93.5%. Thus, radiomics analysis may act as a valid noninvasive auxiliary method to improve the classification of sHCC from benign nodules in cirrhotic liver.

In addition, we found that antiviral therapies may reduce the risk of hepatocarcinogenesis in both HCV and HBV patients and the proportion of patients who received antiviral therapies in the HCC group was lower than that in the benign nodule group, supporting the findings of a previous study in which antiviral therapies were confirmed to play an important role in the prognosis and evolution of HCC [36].

There are several limitations of this study. First, because of the retrospective single-center nature of our study design, these results are preliminary and needed validation using an external dataset to assess their reproducibility and clinical translation. Second, approximately 54.7% of the patients accepted Gd-DTPA-enhanced imaging, so ancillary features based on HBP imaging were unavailable for these cases. In addition, the growth threshold was not considered in the assessment, because MRI follow-up data for more than 6 months were unavailable for most patients. Nevertheless, the diagnostic value of the growth threshold may be low [10], and follow-up of high-risk patients with nodules > 10 mm does not correspond to existing international guidelines [37]. Third, the population of this study was composed largely of HBV patients, and only contained a minority of patients with HCV-related cirrhosis. Thus, a comparison of results between the HBV and HCV groups was not performed. Finally, only small liver lesions were included in this study, because of which the possibility of selection bias may not have been completely avoided.

## Conclusions

MRI-based radiomics analysis may supplement the value of the LI-RADS v 2018 algorithm in the differentiation of small HCC from benign nodules in the cirrhotic liver.

## Supplementary Information

**Additional file 1: Table 1**. Algorithm and diagnostic table based on major imaging features.

**Additional file 2: Table 2**. Ancillary features in LI-RADS.

**Additional file 3: Table 3**. Texture features calculated in MaZda.

## Data Availability

The datasets used and/or analyzed during the current study are available from the corresponding author on reasonable request.

## Abbreviations

HCC: Hepatocellular carcinoma
LI-RADS: Liver imaging reporting and data system
MRI: Magnetic resonance imaging
DWI: Diffusion-weighted MR imaging
ADC: Apparent diffusion coefficient
PPV: Positive predictive value
NPV: Negative predictive value
ROC: Receiver operating characteristic curve
A_Z_: Area under the receiver operating characteristic curve
RT: Radiotherapy
ICC: Intraclass correlation coefficient

## References

[1] Park HJ, Choi BI, Lee ES, Park SB, Lee JB (2017). How to differentiate borderline hepatic nodules in hepatocarcinogenesis: emphasis on imaging diagnosis. Liver cancer.

[2] Di Martino M, Anzidei M, Zaccagna F, Saba L, Bosco S, Rossi M (2016). Qualitative analysis of small (</=2 cm) regenerative nodules, dysplastic nodules and well-differentiated HCCs with gadoxetic acid MRI. BMC Med Imaging.

[3] Mitchell DG, Bruix J, Sherman M, Sirlin CB (2015). LI-RADS (Liver Imaging Reporting and Data System): summary, discussion, and consensus of the LI-RADS Management Working Group and future directions. Hepatology.

[4] Kielar AZ, Chernyak V, Bashir MR, Do RK, Fowler KJ, Mitchell DG (2018). LI-RADS 2017: an update. J Magn Reson Imaging.

[5] Cerny M, Chernyak V, Olivie D, Billiard JS, Murphy-Lavallee J, Kielar AZ (2018). LI-RADS version 2018 ancillary features at MRI. Radiographics.

[6] Chernyak V, Fowler KJ, Kamaya A, Kielar AZ, Elsayes KM, Bashir MR (2018). Liver imaging reporting and data system (LI-RADS) version 2018: imaging of hepatocellular carcinoma in at-risk patients. Radiology.

[7] Kielar AZ, Chernyak V, Bashir MR, Do RK, Fowler KJ, Santillan C (2019). An update for LI-RADS: version: Why so soon after version 2017?. J Magn Reson Imaging.

[8] Renzulli M, Clemente A, Brocchi S, Milandri M, Lucidi V, Vukotic R, Cappabianca S, Golfieri R (2019). LI-RADS: a great opportunity not to be missed. Eur J Gastroenterol Hepatol.

[9] Ronot M, Fouque O, Esvan M, Lebigot J, Aube C, Vilgrain V (2018). Comparison of the accuracy of AASLD and LI-RADS criteria for the non-invasive diagnosis of HCC smaller than 3cm. J Hepatol.

[10] Choi SH, Byun JH, Kim SY, Lee SJ, Won HJ, Shin YM, Kim PN (2016). Liver imaging reporting and data system v2014 with gadoxetate disodium-enhanced magnetic resonance imaging: validation of LI-RADS category 4 and 5 criteria. Invest Radiol.

[11] Joo I, Lee JM, Lee DH, Jeon JH, Han JK (2019). Retrospective validation of a new diagnostic criterion for hepatocellular carcinoma on gadoxetic acid-enhanced MRI: can hypointensity on the hepatobiliary phase be used as an alternative to washout with the aid of ancillary features?. Eur Radiol.

[12] Zhou J, Zhang Y, Chang KT, Lee KE, Wang O, Li J (2020). Diagnosis of benign and malignant breast lesions on DCE-MRI by using radiomics and deep learning with consideration of peritumor tissue. J Magn Reson Imaging.

[13] Ji Y, Li H, Edwards AV, Papaioannou J, Ma W, Liu P, Giger ML (2019). Independent validation of machine learning in diagnosing breast Cancer on magnetic resonance imaging within a single institution. Cancer Imaging.

[14] Ortiz-Ramon R, Larroza A, Ruiz-Espana S, Arana E, Moratal D (2018). Classifying brain metastases by their primary site of origin using a radiomics approach based on texture analysis: a feasibility study. Eur Radiol.

[15] Schieda N, Krishna S, McInnes MDF, Moosavi B, Alrashed A, Moreland R, Siegelman ES (2017). Utility of MRI to differentiate clear cell renal cell carcinoma adrenal metastases from adrenal adenomas. AJR Am J Roentgenol.

[16] Wibmer A, Hricak H, Gondo T, Matsumoto K, Veeraraghavan H, Fehr D (2015). Haralick texture analysis of prostate MRI: utility for differentiating non-cancerous prostate from prostate cancer and differentiating prostate cancers with different Gleason scores. Eur Radiol.

[17] Nketiah G, Elschot M, Kim E, Teruel JR, Scheenen TW, Bathen TF, Selnaes KM (2017). T2-weighted MRI-derived textural features reflect prostate cancer aggressiveness: preliminary results. Eur Radiol.

[18] Li Z, Mao Y, Huang W, Li H, Zhu J, Li W, Li B (2017). Texture-based classification of different single liver lesion based on SPAIR T2W MRI images. BMC Med Imaging.

[19] Mayerhoefer ME, Schima W, Trattnig S, Pinker K, Berger-Kulemann V, Ba-Ssalamah A (2010). Texture-based classification of focal liver xlesions on MRI at 3.0 Tesla: a feasibility study in cysts and hemangiomas. J Magn Reson Imaging.

[20] Holli K, Laaperi AL, Harrison L, Luukkaala T, Toivonen T, Ryymin P (2010). Characterization of breast cancer types by texture analysis of magnetic resonance images. Acad Radiol.

[21] Wu S, Zheng J, Li Y, Yu H, Shi S, Xie W (2017). A radiomics nomogram for the preoperative prediction of lymph node metastasis in bladder cancer. Clin Cancer Res.

[22] Szczypinski PM, Strzelecki M, Materka A, Klepaczko A (2009). MaZda—a software package for image texture analysis. Comput Methods Programs Biomed.

[23] Liu S, He J, Liu S, Ji C, Guan W, Chen L, Guan Y, Yang X, Zhou Z (2020). Radiomics analysis using contrast-enhanced CT for preoperative prediction of occult peritoneal metastasis in advanced gastric cancer. Eur Radiol.

[24] Hou Z, Yang Y, Li S, Yan J, Ren W, Liu J, Wang K, Liu B, Wan S (2018). Radiomic analysis using contrast-enhanced CT: predict treatment response to pulsed low dose rate radiotherapy in gastric carcinoma with abdominal cavity metastasis. Quant Imaging Med Surg.

[25] Huang YQ, Liang CH, He L, Tian J, Liang CS, Chen X (2016). Development and validation of a radiomics nomogram for preoperative prediction of lymph node metastasis in colorectal cancer. J Clin Oncol.

[26] Zhang L, Dong D, Li H, Tian J, Ouyang F, Mo X (2019). Development and validation of a magnetic resonance imaging-based model for the prediction of distant metastasis before initial treatment of nasopharyngeal carcinoma: a retrospective cohort study. EBioMedicine.

[27] Yu H, Buch K, Li B, O'Brien M, Soto J, Jara H, Anderson SW (2015). Utility of texture analysis for quantifying hepatic fibrosis on proton density MRI. J Magn Reson Imaging.

[28] Zhou W, Zhang L, Wang K, Chen S, Wang G, Liu Z, Liang C (2017). Malignancy characterization of hey6patocellular carcinomas based on texture analysis of contrast-enhanced MR images. J Magn Reson Imaging.

[29] Zhong X, Tang H, Lu B, You J, Piao J, Yang P, Li J (2019). Differentiation of small hepatocellular carcinoma from dysplastic nodules in cirrhotic liver: texture analysis based on MRI improved performance in comparison over gadoxetic acid-enhanced MR and diffusion-weighted imaging. Front Oncol.

[30] Stocker D, Marquez HP, Wagner MW, Raptis DA, Clavien PA, Boss A, Fischer MA, Wurnig MC (2018). MRI texture analysis for differentiation of malignant and benign hepatocellular tumors in the non-cirrhotic liver. Heliyon.

[31] Vasuri F, Renzulli M, Fittipaldi S, Brocchi S, Clemente A, Cappabianca S, Bolondi L, Golfieri R, D'Errico A (2019). Pathobiological and radiological approach for hepatocellular carcinoma subclassification. Sci Rep.

[32] Giannini EG, Moscatelli A, Pellegatta G, Vitale A, Farinati F, Ciccarese F, Piscaglia F, Rapaccini GL, Di Marco M, Caturelli E (2016). Application of the intermediate-stage subclassification to patients with untreated hepatocellular carcinoma. Am J Gastroenterol.

[33] Min X, Li M, Dong D, Feng Z, Zhang P, Ke Z (2019). Multi-parametric MRI-based radiomics signature for discriminating between clinically significant and insignificant prostate cancer: cross-validation of a machine learning method. Eur J Radiol.

[34] Lim KS (2014). Diffusion-weighted MRI of hepatocellular carcinoma in cirrhosis. Clin Radiol.

[35] Inchingolo R, De Gaetano AM, Curione D, Ciresa M, Miele L, Pompili M (2015). Role of diffusion-weighted imaging, apparent diffusion coefficient and correlation with hepatobiliary phase findings in the differentiation of hepatocellular carcinoma from dysplastic nodules in cirrhotic liver. Eur Radiol.

[36] Guarino M, Sessa A, Cossiga V, Morando F, Caporaso N, Morisco F (2018). Direct-acting antivirals and hepatocellular carcinoma in chronic hepatitis C: a few lights and many shadows. World J Gastroenterol.

[37] Bruix J, Sherman M (2011). American Association for the Study of Liver D. Management of hepatocellular carcinoma: an update. Hepatology.

