# Supplementary material for: Differentiation of small (≤ 3 cm) hepatocellular carcinomas from benign nodules in cirrhotic liver: the added additive value of MRI-based radiomics analysis to LI-RADS version 2018 algorithm
Source: BMC Gastroenterol. 2021 Apr 7;21:155. doi: 10.1186/s12876-021-01710-y (PMC8028813; doi:10.1186/s12876-021-01710-y)
Supplement: Supplementary file 1 — Additional file 1: Table 1. Algorithm and diagnostic table based on major imaging features. [file 12876_2021_1710_MOESM1_ESM.docx]

**Supplementary Table 1. Algorithm and Diagnostic Table Based on Major Imaging Features**

| **APHE** |  | **No APHE** | | **Nonrim APHE** | | |
| --- | --- | --- | --- | --- | --- | --- |
| Lesion size |  | <20mm | ≥20mm | <10mm | 10-19mm | ≥20mm |
| Count additonal features: | None | LR-3 | LR-3 | LR-3 | LR-3 | LR-4 |
| Enhancing “capsule” | One | LR-3 | LR-4 | LR-4 | LR-4/LR-5^#^ | LR-5 |
| Nonperipheral “washout” | ≥ Two | LR-4 | LR-4 | LR-4 | LR-5 | LR-5 |

*APHE* arterial phase hyperenhancement, ^#^*LR-4*, if enhancing “capsule”; LR-5, if nonperipheral “washout”
