# Supplementary material for: Differentiation of small (≤ 3 cm) hepatocellular carcinomas from benign nodules in cirrhotic liver: the added additive value of MRI-based radiomics analysis to LI-RADS version 2018 algorithm
Source: BMC Gastroenterol. 2021 Apr 7;21:155. doi: 10.1186/s12876-021-01710-y (PMC8028813; doi:10.1186/s12876-021-01710-y)
Supplement: Supplementary file 2 — Additional file 2: Table 2. Ancillary features in LI-RADS. [file 12876_2021_1710_MOESM2_ESM.docx]

**Supplementary Table 2. Ancillary Features in LI-RADS**

| **Favoring malignancy in general** | **Favoring HCC in particular** | **Favoring benignity** |
| --- | --- | --- |
| Corona enhancement | Nonenhancing “capsule” | Parallels blood pool enhancement |
| Fat sparing in solid mass | Mosaic architecture | Undistorted vessels |
| Restricted diffusion | Nodule-in-nodule architecture | Marked T2 hyperintensity |
| Mild-moderate T2 hyperintensity | Fat in mass, more than in adjacent liver | HBP isointensity |
| Transitional phase hypointensity | Blood products in mass |  |
| Hepatobiliary phase (HBP) hypointensity |  |  |
