# Supplementary material for: Differentiation of small (≤ 3 cm) hepatocellular carcinomas from benign nodules in cirrhotic liver: the added additive value of MRI-based radiomics analysis to LI-RADS version 2018 algorithm
Source: BMC Gastroenterol. 2021 Apr 7;21:155. doi: 10.1186/s12876-021-01710-y (PMC8028813; doi:10.1186/s12876-021-01710-y)
Supplement: Supplementary file 3 — Additional file 3: Table 3. Texture features calculated in MaZda. [file 12876_2021_1710_MOESM3_ESM.docx]

**Supplementary Table 3. Texture features calculated in MaZda**

| **Statistical methods** | **Texture features** | |
| --- | --- | --- |
| Histogram (n=9) | Mean, Skewness, Variance, Kurtosis, Percentiles 1%, 10%, 50%, 90%, and 99% | |
| Gray-level co-occurrence matrix (n=220) | Angular second moment, Contrast, Difference entropy, Difference variance, Entropy, Correlation, Sum of squares, Inverse difference moment, Sum average, Sum variance, and Sum entropy. Features are calculated for 5 between-pixels distances (1 - 5) and for 4 directions (0, vertical, horizontal, 135). |  |
| Run-length matrix (n=20) | Run-length nonuniformity, Long-run emphasis, Gray-level nonuniformity, Short run emphasis, and Fraction of image in runs .Features are calculated for 4 directions (0, vertical, horizontal, 135). | |
| Wavelet (n=20) | Wavelet energy. Features are calculated at 5 scales within 4 frequency bands HL, HH, LL and LH. | |
| Autoregressive model (n=5) | Teta1 ,Teta2, Teta3 ,Teta4 and Sigma | |
| Absolute gradient (n=5) | Mean, Variance, Skewness, Kurtosis, and Percentage of pixels with nonzero gradient | |
|  |  | |

|  |  |
| --- | --- |
